# Can the velocity profile in the bench press and the bench pull sufficiently estimate the one repetition maximum in youth elite cross-country ski and biathlon athletes?

**DOI:** 10.1186/s13102-025-01137-y

**Published:** 2025-04-28

**Authors:** Carl-Maximilian Wagner, Michael Keiner, Sebastian Puschkasch-Möck, Klaus Wirth, Stephan Schiemann, Konstantin Warneke

**Affiliations:** 1 Institute of Exercise, Sport and Health, Leuphana University Lüneburg, Lüneburg, Germany; 2 Department for Training Science, German University of Health and Sport, Ismaning, Germany; 3 Department of Exercise Science, Olympic Training and Testing Center of Hessen, Frankfurt am Main, Germany; 4 University of Applied Sciences Wiener Neustadt, Wiener Neustadt, Austria; 5 Department for Human Movement Science and Exercise Physiology, Friedrich Schiller University Jena, Jena, Germany

**Keywords:** Maximum strength, Elite athletes, Prediction, Reliability, Measurement

## Abstract

**Introduction:**

In recent years, load-velocity profiles (LVP) have been frequently proposed as a highly reliable and valid alternative to the one-repetition maximum (1RM) for estimating maximal strength and prescribing training loads. However, previous authors commonly report intraclass correlation coefficients (ICC) while neglecting to calculate the measurement error associated with these values. This is important for practitioners, especially in an elite sports setting, to be able to differentiate between small but significant changes in performance and the error rate.

**Methods:**

49 youth elite athletes (17.71±2.07 years) were recruited and performed a 1RM test followed by a load-velocity profiling test using 30%, 50% and 70% of the 1RM in the bench press and bench pull, respectively. Reliability analysis, ICCs and the coefficient of variability, were calculated and supplemented by an agreement analysis including the mean absolute error (MAE) and mean absolute percentage error (MAPE) to provide the resulting measurement error. Furthermore, validity analyses between the measured 1RM and different calculation models to estimate 1RM were performed.

**Results:**

Reliability values were in accordance with current literature (ICC = 0.79–0.99, coefficient of variance [CV] = 1.86–9.32%), however, were accompanied by a random error (mean absolute error [MAE]: 0.05–0.64 m/s, mean absolute percentage error [MAPE]: 2.7–9.5%) arising from test-retest measurement. Strength estimation via the velocity-profile overestimated the bench pull 1RM (limits of agreement [LOA]: -9.73 – -16.72 kg, MAE: 9.80–17.03 kg, MAPE 16.9–29.7%), while the bench press 1RM was underestimated (LOA: 3.34–6.37 kg, MAE: 3.74–7.84 kg, MAPE: 7.5–13.4%); dependent on used calculation model.

**Discussion:**

Considering the observed measurement error associated with LVP-based methods, it can be posited that their utility as a programming strategy is limited. The lack of accuracy required to discriminate between small but significant changes in performance and error, coupled with the potential risks of under- and overestimating 1RM, can result in insufficient stimulus or increased injury risk, respectively. This further diminishes the practicality of these methods, particularly in elite sports settings.

## Introduction

Maximal strength is considered a critical component of athletic performance in a multitude of sports disciplines [1]. In cross-country skiing and the skiing segment of biathlon, upper-body strength has become a particularly crucial element [2–4]. Recent advancements in these sports, including the introduction mass starts and sprint events, have led to higher competition speeds and elevated the importance of techniques like G3 skating and double poling [3, 5]. These high-speed techniques rely heavily on poling for propulsion, necessitating that athletes produce considerable upper-body maximal strength and power to generate high peak forces within short contact times. This enhances the cycle length, which ultimately contributes to increase maximal velocities and competitive success [3, 4, 6]. The ability to exert high muscle forces is commonly improved via resistance training, with training intensity typically being quantified in relation to maximal strength [7]. Accordingly, upper-body pulling and pushing exercises, such as the bench press and bench pull, have become fundamental in high-performance training programs, to facilitate the necessary neural and morphological strength adaptations [8]. In accordance with the current literature, intensities ranging from 80 to 100% of the dynamic maximal strength are recommended when aiming to improve the maximal strength strength-trained athletes [7, 9, 10]. To ensure sufficient load and load progression (2–10%) to optimally stimulate muscular adaptions without an increased risk for injury, an accurate assessment of maximal strength is imperative [11, 12].

The One-Repetition Maximum (1RM) test has historically been regarded as the “gold standard” for assessing dynamic maximal strength. The term is defined as the maximal weight that can be lifted in a single repetition, while maintaining proper lifting technique [12]. In general, the 1RM is considered as a reliable method for estimating maximum strength in adults, with reported ICCs ranging from 0.64 to 0.99, with a median value of 0.97, and 92% of the ICCs reported higher than 0.9 [13, 14]. Nevertheless, those who are critical of 1RM tests argue that maximum strength values are subject to considerable variability, contingent on the athlete’s daily form or a lack of familiarity with 1RM testing in the intended exercises [15]. Indeed, to ensure reliable and safe 1RM testing, it appears essential to include strength-trained participants to meet the requirements for reliable and valid maximal strength estimations via the 1RM testing [11]. Furthermore, practical limitations have been identified in large group settings [16, 17], with concerns that inadequate supervision and limited time capacity may lead to incorrect movement execution at maximal loads, as well as infrequent strength testing, thereby increasing the risk of injury [17, 18].

In recent years, velocity-based training (VBT) methods have been proposed as an accessible, accurate, and precise alternative to overcome the limitations associated with traditional 1RM testing [16, 18–21]. Central to VBT is the assumption of a linear, inverse relationship between load and movement velocity, known as the load-velocity profile (LVP), which extends to terminal velocity at maximum load [16, 22, 23]. By employing technology (e.g., linear position transducers, optical motion sensing systems, accelerometers) VBT tracks bar displacement velocity during exercises to extrapolate 1RM and autoregulate exercise intensity and volume in real-time, accounting for neuromuscular fatigue through velocity loss [20, 24, 25]. Consequentially, this approach warrants precise and accurate velocity measurements, with sufficient sensitivity and minimal bias, to detect small yet meaningful changes in the biological system [23, 26]. VBT methods demonstrate high reliability (ICC = 0.65–0.99) for resistance training load control across various populations [19, 22, 27–29]. Other studies have generally supported the use of LVPs to estimate 1RM and %1RM across various exercises [22, 30–32], though evidence suggests that LVPs are exercise-specific [16, 24, 32], may vary by sex [33], and are influenced by individual factors such as biomechanics, muscle fiber type, and therefore training history [34–38]. However, most research has been conducted on small samples (*n* = 6 to 30) of recreationally trained males, limiting its applicability to elite sports settings. Given the suggested benefits of VBT, several authors have recommended incorporating VBT and LVPs into diagnostic and daily training practice [28, 39]. However, Goldsmith, Trepeck [40] caution against potential pitfalls, such as inappropriate statistical modeling or the lack of a true criterion for agreement assessment in previous literature, which may limit the practical utility of VBT for exercise prescription and autoregulation due to significant measurement errors and poor agreement.

To the best of the authors’ knowledge, to date no study investigated LVPs and extrapolated 1RMs within a setting of highly trained endurance athletes. However, due to the growing relevance of upper body strength and power capacity in cross-country skiing and biathlon and VBT approaches entering performance diagnostics in the elite sports domain [21], this research gap presents practical interest. Accordingly, this study explores relative reliability values arising from test-retest data collection and additionally provides values for the systematic and random error. Furthermore, the work compares the measured 1RM value from maximal dynamic strength testing with the calculated value using suggested velocity thresholds extracted from literature [41]. Only the quantification of the magnitude of measurement errors enables the differentiation between practical significant changes in performance between repeated trails and error rate. Due to advocated high practical relevance of velocity based maximal strength estimation, a strong agreement with minimal measurement error arising from test-retest reliability as well as the estimated maximal strength versus the measured 1RM can be hypothesized.

## Methods

### Participants

Forty-nine national-level (Tier 3 [42]) youth cross-country skiers and biathletes (male, *n* = 25; female, *n* = 24; age: 17.71±2.07 years; height: 1.73±0.1 m, body mass: 62.36±9.56 kg, VO2max: 65.7 ± 6.8 ml•kg^− 1^•min^− 1^) insuring adequate sample size were recruited from two high-performance training centers [43]. Participants were consistently ranked among the top 30 in their respective national and international competition classes and can therefore be defined as elite [44]. In addition to their endurance training (6–12 session for 8.5–16.5 h training/week), the participants performed 1–2 resistance training sessions per week regularly (1.5–3 h training/week). Their respective strength training experiences ranged from 0.5 to 4 years. None of the participants reported any injuries at the time of testing. Participants and their parents (if the participants were younger than 18 years) were informed about the study’s objectives and possible risks and provided written informed consent. This study was performed in accordance with the Helsinki Declaration and was approved by the Universities Ethics Committee (German University of Health and Sport, DHGS-EK-2023-004).

### Testing procedure

The assessment protocol comprised the determination of 1RM in both bench press and bench pull followed by maximal power attempts at submaximal loads to set up the corresponding load-velocity profile. The testing order for bench press and bench pull remained the same during the complete protocol. Verbal encouragement was provided to athletes throughout all tests to stimulate maximal effort. All participants were experienced in performing the included resistance training exercises as part of their regular training routine.

One week prior to the testing session the participants performed the following routine to estimate their current 1RM: Three sub-maximal sets, with increasing load: 10 repetitions at approx. 40%, 6 repetitions at approx. 75%, and 3 repetitions at approx. 85% of the estimated 1RM from training loads. Then, two heavier, almost maximal lifts were performed to estimate 1RM.

To minimize potential confounding factors, participants exercised for a maximum of ninety minutes at low intensities (< 75% of maximum heart rate) the day before the testing. They were instructed to avoid eating and consuming coffee, or other products containing caffeine during the last 2 to 3 h before testing. On test days, participants refrained from performing any training before testing.

### Maximal strength determination via 1RM

1RM testing in both bench press and bench pull was conducted in accordance with the guidelines established by the National Strength and Conditioning Association [45]. A standardized protocol was conducted, including 10-minute warm-up on a cycle ergometer, followed by multiple repetitions with submaximal loads (3 sets of 6–8 repetitions at 50–80% of 1RM for each exercise). The initial attempts for both exercises involved a load of approximately 90–95% of the estimated 1RM. After each successful attempt, the load was increased by 2–5% until participants were unable to press or pull the load with proper technique. Following two consecutive unsuccessful attempts, the highest accepted attempt was considered for further calculation. During all attempts the participants were instructed to perform the concentric phase of the exercise with maximal acceleration and speed. Rest periods of at least 5 min were observed between trials. 1RMs were determined within a maximum of 5 attempts. All 1RM testing was supervised by the same investigator and conducted on the same equipment with identical equipment positioning for each subject.

Correct movement execution required a 5-point body contact position which included maintaining firm contact with the head, upper back, and buttocks on the bench while keeping both feet flat on the ground. During the eccentric phase, gentle contact of the barbell with the chest was permitted. If the chest movement aided the execution, the attempt would be considered unsuccessful. The end position was defined by fully extended elbows at the end of the concentric movement [46].

In the prone bench pull, the examiner visually ensured confirmed straight arm positioning as participants grasped the barbell. Arm flexion was initiated from the extended position and the movement was considered finalized when the barbell touched the bench with an elbow angle of ≤ 90° [47]. For a successful trial, the chest and lower extremities were required to maintain in contact with the bench.

### Load-velocity profile with submaximal loads

The LVP was determined using loads corresponding to 30%, 50%, and 70% of the previously determined 1RM. This selection was chosen based on prior literature suggesting that maximal power output may manifest within this range [30, 31]. During power testing, participants were instructed to exert maximum force and speed during the concentric phase of the respective movement. A brief rest period (approximately 1 s) followed the eccentric and preceded the concentric muscle actions to prevent coupling between eccentric and concentric movements. Two concentric actions were recorded for each load. The rest interval between each trial was 2 min. Bar displacement, mean concentric velocity, and both peak and mean concentric power were captured by attaching a rotary encoder (Tendo Unit, TENDO Sport, Trencin, Slovak Republic) to one end of the barbell. The rotary encoder precisely recorded the position and direction of the bar, with an accuracy of 0.3 mm. Customized software was employed to compute power output for each repetition of the bench press performed throughout the range of motion. The TENDO Unit device was described a reliable device when measuring average and peak bar velocities [32].

### 1RM calculation based on the velocity profile

Based on the velocity profile at 30%, 50% and 70% of the 1RM the estimated 1RM was approximated as described in Hughes et al. (2019). The minimal velocity threshold calculation for the bench press and pull were performed with v = 0.1 m/s and v = 0.2 m/s [14, 33], while assuming the movement velocity to be minimal (near 0 m/s). Accordingly, the velocity was set to zero to estimate the 1RM via the load at zero estimation (LD0).

### Statistical analyses

The Data analysis was performed using SPSS 28.0 (IBM, Ehningen, DE, Germany). The descriptive statistics of the 1RM weight from each trial, submaximal loads (30%, 50%, 70% 1RM) as well as the respective average velocity and peak velocity were provided via mean (M) ± standard deviation (SD). The significance level for all statistical tests was set at *p* < 0.05. Data normal distribution was ensured using the Shapiro Wilk test.

To assess *intra-day reliability*, intraclass correlation coefficients (ICCs) with 95% confidence intervals (CIs) and coefficients of variation (CVs) were calculated. Reproducibility, which requires a high degree of agreement, was analyzed in this study using statistical methods to quantify measurement error associated with ICCs. Therefore, Bland-Altman (BA) analyses were conducted [28, 29]. While BA analysis primarily provides a visual inspection of agreement by plotting systematic error alongside the lower and upper limits of agreement, further quantification of measurement error beyond the 95% CI limits offers additional value. Specifically, the mean absolute error (MAE) [37, 38] quantifies the magnitude of errors between paired observations measuring the same parameter, while the mean absolute percentage error (MAPE) [39–41] represents accuracy by indicating the relative deviation between two measurement procedures [8].

To assess *validity*, the estimated maximal strength (derived from the velocity profile at 30%, 50%, and 70% 1RM) was compared with the measured maximal strength (1RM). A paired t-test was conducted to calculate the systematic bias between the estimated and measured 1RM. Additionally, a Bland-Altman (BA) plot was generated, including the lower and upper limits of agreement. Furthermore, both the mean absolute error (MAE) and the mean absolute percentage error (MAPE) were calculated to provide deeper insights into the agreement and accuracy of the measurements. The standard error of difference (SED) was also determined to assess the precision of the estimated 1RM relative to the measured values.

## Results

### Reliability

Reliability statistics (test-retest, intra-day), including average and peak power, average and peak velocity, and peak force, are presented in Table 1. Overall, the reliability values ranged from moderate to high, with intraclass correlation coefficients (ICC) ranging from 0.82 to 0.97 for the bench pull and from 0.65 to 0.85 for the bench press and coefficients of variation (CV) ranging from 1.86 to 6.72%.

**Table 1:** Intraday Test-Retest reliability of velocity measurements across relative 1RM loads using a linear position transducer: baseline for data processing

Parameter	ICC (95% CI)	CV (in%)	Agreement bias between first and second trial (95% CI)	Lower – upper limit of agreement	MAE	MAPE (in%)
**Bench Pull**						
AV (30%1RM)	0.845 (0.740–0.909)	5.44±6.9	-0.007 (-0.044–0.031)	-0.27–0.25	0.12	7.1%
PV (30%1RM)	0.850 (0.749–0.913)	5.13±5.9	0.04 (-0.014–0.093)	-0.32–0.40	0.13	7.1%
AV (50%1RM)	0.906 (0.838–0.946)	3.29±3.1	0.001 (-0.018–0.020)	-0.13–0.13	0.064	6.6%
PV (50%1RM)	0.974 (0.954–0.985)	1.86±1.9	-0.002 (-0.017–0.013)	-0.10–0.10	0.047	2.7%
AV (70%1RM)	0.822 (0.703–0.896)	4.74±7.6	-0.0042 (-0.025–0.024)	-0.165–0.164	0.60	8.9%
PV (70%1RM)	0.957 (0.925–0.976)	3.11±2.3	0.003 (-0.012–0.002)	-0.1–0.1	0.06	6.3%
**Bench Press**						
AV (30%1RM)	0.734 (0.570–0.841)	3.84±4.2	-0.024 (-0.05–0.00057)	-0.19–0.14	0.64	5.7%
PV (30%1RM)	0.650 (0.453–0.787)	4.26±4.9	0.008 (-0.04–0.05)	-0.30–0.32	0.11	5.9%
AV (50%1RM)	0.851 (0.749–0.913)	4.28±8.9	0.00083 (-0.013–0.015)	-0.093–0.095	0.034	4.4%
PV (50%1RM)	0.853 (0.754–0.915)	3.59±3.5	-0.012 (-0.04–0.012)	-0.18–0.15	0.08	7.16%
AV (70%1RM)	0.835 (0.726–0.903)	6.38±5.6	0.009 (-0.007–0.25)	-0.1–0.12	0.04	8.8%
PV (70%1RM)	0.788 (0.654–0.874)	6.72±6.4	0.011 (-0.017–0.04)	-0.18–0.20	0.07	9.5%

ICC, intraclass correlation coefficient; 95%CI, 95% confidence interval; CV, coefficient of variation; MAE, mean absolute error; MAPE, mean absolute percentage error; AV, average velocity in m·s^− 1^; PV, peak velocity in m·s^− 1^; %1RM, percentage of measured one-repetition maximum

### Validity

**Fig. 1:**
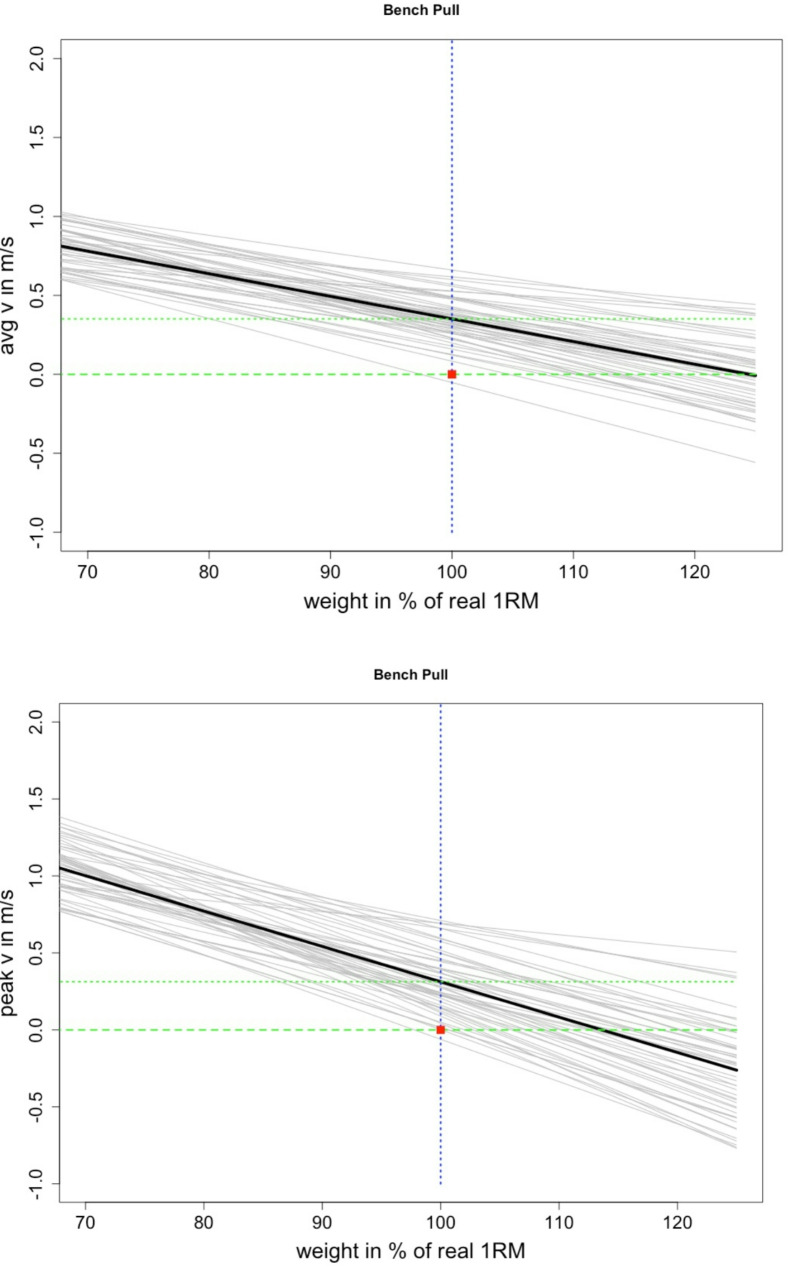
Graphical illustration of the measured 1RM bench pull (red dot), estimated 1RM (regression crossing the 0 line) and calculated movement velocity at the measured 1RM (100%) using the average and peak velocity (20) as well as the deviation of the measurement error, in percentage. The dotted green line crossing the vertical blue dotted line shows the actual mean velocity at 100% of the 1RM to graphically illustrate the discrepancy between the actually measured and the estimated 100% 1RM

**Fig. 2:**
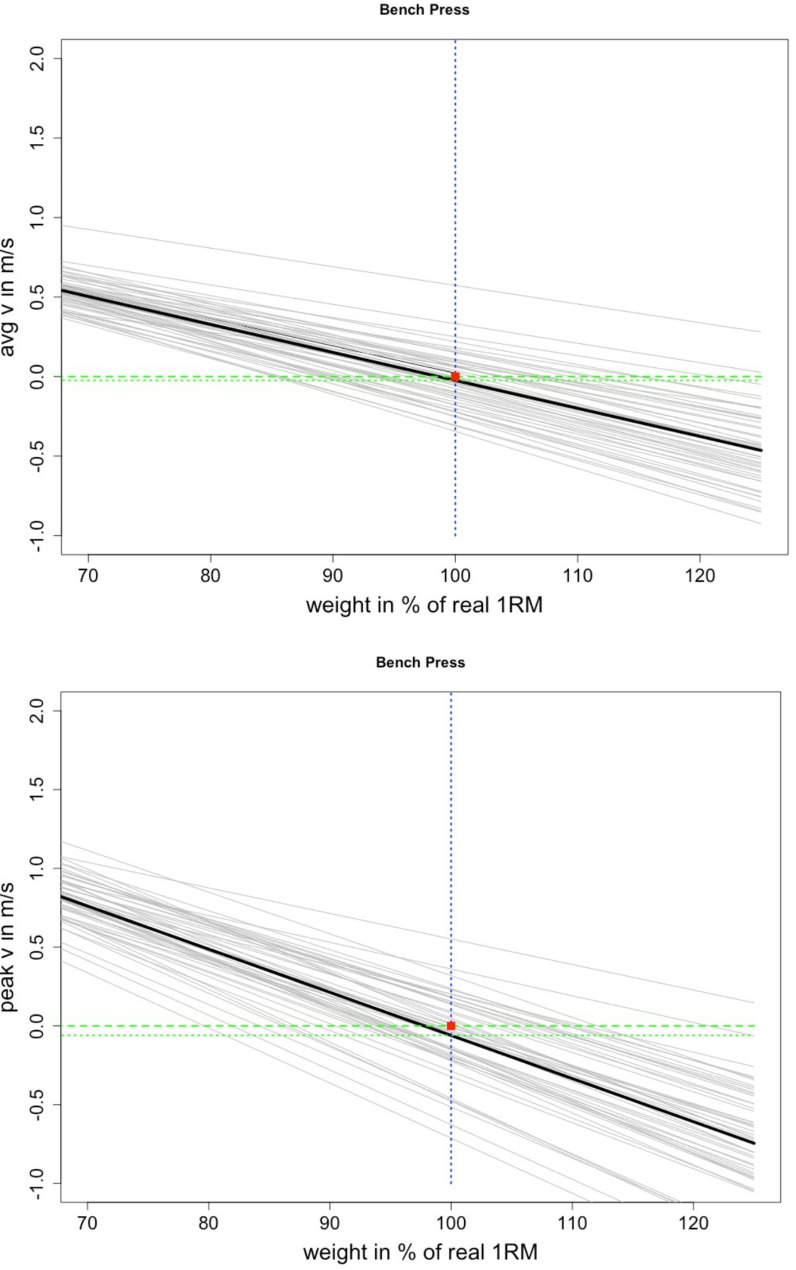
Graphical illustration of the measured 1RM bench press (red dot), estimated 1RM (regression crossing the 0 line) and calculated movement velocity at the measured 1RM (100%) using the average and peak velocity as well as the deviation of the measurement error, in %. The dotted green line crossing the vertical blue dotted line shows the actual mean velocity at 100% of the 1RM to graphically illustrate the discrepancy between the actually measured and the estimated 100% 1RM

The analysis of validity using different velocity-based methods revealed distinct trends for the bench pull and bench press exercises (Table 2; Figs. 1 and 2). For the bench pull, the estimated 1RM was consistently overestimated across most conditions, with systematic biases and error rates indicating significant overprediction. In contrast, the bench press demonstrated a general trend of underestimation, with calculated 1RM values systematically lower than the actual measured values. Furthermore, the MAE and MAPE for both exercises were notably high.

**Table 2:** Agreement analysis of measured vs. Predicted 1RM using the load.Velocity relationship: comparison of MVT and LD0 methods across two trials per relative load

Parameter	Trial	1RM (in kg)	P1RM (in kg)	Sig.	Systematic bias (95% CI) (in kg)	Lower – upper limit of agreement (in kg)	MAE (in kg)	MAPE (in %)	SED
**Bench Pull MVT (0.1 m·s** ^− **1**^ **)**									
AV	1	59.9±17.8	72.1±22.7	*p* < 0.001	-12.2 (-15.32 – -9.09)	-33.19–8.8	12.40	21.21	1.55
	2	59.9±17.8	72.0± 22.1	*p* < 0.001	-12.4 (-15.11 - − 9.70)	-30.87–6.06	12.81	22.6	1.35
PV	1	59.9±17.8	66.6±20.8	*p* < 0.001	-7.01±-9.62 – -4.41	-24.80–10.77	7.41	13.0	1.3
	2	59.9±17.8	67.31±19.5	*p* < 0.001	-7.72 (-9.79 – -5.66)	-21.83–6.38	6.38	8.02	1.03
**Bench Press MVT (0.1 m·s** ^**− 1**^ **)**									
AV	1	52.9±18.9	49.6±17.3	*p* < 0.001	3.34 (2.75 – -4.92)	-7.49–14.16	5.07	9.6	0.789
	2	52.9±18.9	48.1±16.7	*p* < 0.001	4.85 (3.6–6.1)	-3.69–13.39	5.49	10.3	0.62
PV	1	52.9±18.9	50.7	*p* = 0.004	2.22 (0.74–3.70)	-7.86–12.29	4.21	8.3	0.73
	2	52.9±18.9	50.2±18.1	*p* = 0.007	2.7 (0.79–4.60)	-10.32–15.71	4.89	9.13	0.95
**Bench Pull MVT (0.2 m·s** ^**− 1**^ **)**									
AV	1	59.9±17.8	67.7±21.2	*p* < 0.001	-8.12 (-10.77 - -5.47)	-26.22–9.97	8.76	15.4	1.32
	2	59.9±17.8	67.7± 21.1	*p* < 0.001	-8.09 (-10.50 - − 5.68)	-37.30–3.85	16.20	29.6	1.50
PV	1	59.9±17.8	63.9±20.1	*p* < 0.001	-4.3 (-6.64 - -1.96)	-20.27–11.67	5.60	9.9	1.16
	2	59.9±17.8	64.55±19.1	*p* < 0.001	-4.96 (-6.88 – -3.04)	-18.04–8.12	5.82	10.60	0.95
**Bench Press MVT (0.2 m·s** ^**− 1**^ **)**									
AV	1	52.9±18.9	46.6±16.4	*p* < 0.001	6.37 (4.76 – -7.98)	-4.62–17.37	7.23	13.4	0.801
	2	52.9±18.9	45.2±15.9	*p* < 0.001	7.71 (6.35–9.06)	-1.52–16.93	7.84	14.5	0.67
PV	1	52.9±18.9	48.7±18.5	*p* < 0.001	4.21 (2.81 – -5.61)	-5.34–13.77	5.12	9.9	0.70
	2	52.9±18.9	48.3±17.4	*p* < 0.001	4.67 (2.85–6.49)	-18.04–8.12	5.93	11.0	0.95
**Bench Pull LD0 (m·s** ^**− 1**^ **)**									
AV	1	59.9±17.8	76.56± 23.8	*P* < 0.001	-16.96 (-20.57 – -13.4)	-41.59–7.66	17.03	29.7	1.8
	2	59.9±17.8	76.31±23.0	*p* < 0.001	-16.72 (-19.74 – -13.71)	-37.30–3.85	16.2	29.6	1.5
PV	1	59.9±17.8	69.3± 21.6	*p* < 0.001	-9.73 (-12.61 – -6.65)	-29.38–9.93	9.80	16.90	1.43
	2	59.9±17.8	70.01±20.0	*p* < 0.001	-10.49 (-12.71 – -8.26)	-25.66–4.69	10.63	19.20	1.11
**Bench Press LD0 (m·s** ^**− 1**^ **)**									
AV	1	52.9±18.9	52.6±18.3	*p* = 0.712	0.29 (-1.32–1.91)	-10.72–11.32	4.08	8.30	0.30
	2	52.9±18.9	50.9±17.6	*p* = 0.001	2.0 (0.81–3.19)	-6.14–10.14	3.74	7.5	0.593
PV	1	52.9±18.9	52.7±20.1	*p* = 0.778	0.22 (-1.36–1.81)	-10.59–11.04	3.87	7.50	0.788
	2	52.9±18.9	52.2±	0.475	-10.49 (-12.71 – -8.26)	-25.66–4.69	4.30	8.4	1.0

1RM, one-repetition maximum; P1RM, predicted one-repetition maximum; Sig., significance; 95%CI, 95% confidence interval; MAE, mean absolute error; MAPE, mean absolute percentage error; SED, standard error difference; MVT, minimal velocity threshold; LD0, load which corresponds with a velocity of 0 m·s^− 1^; AV, average velocity in m·s^− 1^; PV, peak velocity in m·s^− 1^

## Discussion

Maximum strength is an integral component of elite training programs and are increasingly emphasized in endurance sports such as cross-country skiing and biathlon. It is therefore crucial for athletes and coaches to employ reliable and valid tools with minimal measurement error to estimate 1RM and monitor training load. This is particularly pertinent in elite sport settings, where minor discrepancies in performance can often prove decisive in determining success or failure [48]. The objective of this study was to evaluate the validity and reliability of maximal strength estimation using LVPs in endurance-trained athletes during the bench press and bench pull exercises.

### Reliability

The results of this study demonstrate good to excellent reliability for performance monitoring across a range of loads, with ICC values ranging from 0.65 to 0.97 and CV values between 1.86% and 6.72%, all below 10% for both average and peak power. These findings corroborate those of previous research, which has validated the efficacy of several velocity measurement devices for the assessment of both average and peak velocities [28–30, 49]. The results in previous studies demonstrate moderate to good intraday and interday reliability, respectively, as evidenced by ICCs ranging from 0.55 to 1 and CVs between 1.7% and 11%. Consequently, previous studies have recommended the application of movement velocity measurements for the regulation of training load [27, 28, 39]. Feuerbach et al. highlight, that the reliability of these measurements within sessions contrasts with the moderate interday reliability, necessitating careful interpretation when comparing performance across multiple days. However, practical recommendations for the use of LVPs must take into account the potential for measurement errors, as high levels of consistency and reproducibility are essential for their effective implementation by coaches and athletes [50]. Reproducibility requires a high degree of agreement [51], yet none of these investigations applied appropriate statistical models to evaluate agreement [52–55]. In accordance with statistical research [49–51], the agreement analysis was performed to express the measurement error of the corresponding ICCs. While the majority of ICCs in the present study were classified as high to excellent [56], the corresponding velocity measurement errors exhibited considerable variability with MAEs ranging from 0.047 to 0.64 m/s, contingent on the intensity, with an MAPE of 2.7–9.5% (Table 1). This variability underscores the shortcomings of relying solely on ICC values to assess reliability. The inconsistency renders load-velocity profiling methods impractical for precise and effective training load regulation. High error rates diminish the precision needed for reliable, day-to-day adjustments, posing significant challenges for the practical application of load-velocity profiles [49]. Consequently, movement velocity is unsuitable for monitoring changes in velocity (e.g., for fatigue monitoring) during free-weight bench press and bench pull exercises.

### Validity - Predicting the 1RM via velocity profile

The study examined the validity of various velocity-based methods for estimating 1RM and identified notable trends for both the bench pull and bench press, respectively. In the bench pull, 1RM estimates were found to be consistently overestimated across most conditions, with systematic biases ranging from 4.3 to 16.96 kg. In contrast, the bench press exhibited a consistent pattern of underestimation, with the predicted 1RM values consistently falling below the measured values by 0.22 to 10.49 kg. Bland-Altman analyses revealed the presence of substantial unsystematic errors, which resulted in substantial over- and underestimations across individual subjects. The magnitude of these errors, as evidenced by a high MAE of 3.74 to 17.03 kg and a high MAPE of 7.5–29.7%, underscores the limitations and variability of these predictive models. These limitations, which are not accounted for when reporting means, correlations, and ICCs, have the potential to significantly impact the practical application of LVPs in daily athletic training. The limited validity of focusing on regression models when predicting one parameter by extrapolating submaximal values of another is underscored by Figs. 1 and 2, which graphically illustrate the dispersion of the estimating error.

This study, conducted with elite endurance-trained athletes exhibiting relatively low strength levels (bench pull: 59.9 ± 17.8; bench press: 52.9 ± 18.9), revealed unsystematic measurement errors and poor agreement between predicted and measured 1RM values, aligning with findings from strength-trained individuals [32, 41, 57–59]. The precision and stability of predictive models thereby appear unaffected by strength levels or training backgrounds [16, 30, 32, 60], provided participants are adequately familiarized with testing protocols to exert maximal force [33]. Controversially, Hughes, Banyard [41] reported that discrepancies between predicted and actual 1RM values increase with higher strength levels. Moreover, the force-velocity relationship is influenced by exercise variation and may be affected by execution technique, further complicating prediction accuracy [57]. The non-ballistic nature of movements like the bench press and bench pull poses a fundamental limitation to 1RM estimation via LVPs. Participants may limit maximal acceleration to avoid throwing the barbell, losing contact with the bench, or crashing the bar into the bench, which restricts the recruitment and firing frequency of fast-contracting muscle fibers. This results in underestimated velocity outputs, especially at lower loads, and underestimates the true capability of fast-contracting fibers, leading to inaccuracies in velocity-based 1RM prediction. This may also explain why LVPs constructed with loads closer to the 1RM show somewhat improved precision [58, 61]. Another substantial challenge to accurately predict 1RM via LVPs is the presence of horizontal oscillations during free-weight exercises. Since linear position transducers rely on precise perpendicular alignment to measure vertical displacement, these oscillations can distort measurements, leading to errors in calculated bar velocities and reduced reliability of LVP-based predictions [62–64]. Although some authors have advocated for general and linear regression models as quick and practical methods for estimating %1RM [16, 32, 65, 66], the current results do not support these recommendations. In this study, only 3 out of 24 estimated 1RMs were not significantly different from the actual measured values, and neither average nor peak bar velocity reliably predicted 1RM.

### Limitations

This study provides valuable insights but is subject to several limitations. First, bar displacement measurements were not validated against gold-standard technologies like 3D motion analysis [40], making it difficult to distinguish between device-specific and human-induced errors. Such validation is essential, as systematic differences between devices can increase prediction errors when using generalized regression models. Second, the study relied on generalized regression models derived from the tested population to estimate 1RM. While the velocity ranges for average and peak velocity aligned with prior studies, discrepancies in individual load-velocity profiles suggest that individualized regressions could significantly enhance 1RM estimation accuracy [33]. Third, the use of a general MVTs instead of exercise-specific MVTs likely contributed to variability and reduced predictive accuracy, limiting the applicability of the findings, particularly for lower-body exercises common in endurance athletes [67, 68]. Finally, the study only involved a single testing session, restricting reliability analyses to intra-day measures and omitting inter-day reliability assessments. However, if a method doesn’t provide sufficient reliability within a session (i.e., minimum threshold), it might be obsolete to calculate other measures of reliability and validity.

### Practical applications

The results of this study highlight the limited practical utility of LVP-based methods for exercise prescription and training load adjustment. While this concept was originally proposed to enable autoregulation and optimize training load adjustments for improved performance, the high variability in predictions, coupled with the risk of under- or overestimating 1RM - potentially leading to insufficient training stimulus or increased risk of injury - renders these methods unreliable for such purposes. Furthermore, as LVP-based predictions are unlikely to detect small changes in maximal strength (< 5–7%) [42], their application in elite sport settings is particularly limited. Consequently, this study supports the conclusions of Guppy, Kendall [69] - periodized approaches based on periodic 1RM testing and adjustments to training volume or intensity, remain more effective for strength-trained individuals. Moreover, although LVP-based predictions often show strong correlations with actual 1RM values [41, 58, 61], correlation coefficients fail to address for bias and agreement, highlighting the need for more robust statistical methodologies in future research.

## Data Availability

The datasets used and/or analysed during the current study are available from the corresponding author on reasonable request.

## References

[1] Suchomel TJ, Nimphius S, Stone MH. The importance of muscular strength in athletic performance. Sports Med. 2016;46(10):1419–49. 26838985 10.1007/s40279-016-0486-0

[2] Stoggl T, Holmberg HC. A systematic review of the effects of strength and power training on performance in Cross-Country skiers. J Sports Sci Med. 2022;21(4):555–79. 36523891 10.52082/jssm.2022.555 PMC9741725

[3] Stoggl T, et al. General strength and kinetics: fundamental to sprinting faster in cross country skiing? Scand J Med Sci Sports. 2011;21(6):791–803. 20492588 10.1111/j.1600-0838.2009.01078.x

[4] Wagner C-M, et al. High-Volume resistance training improves Double-Poling peak oxygen uptake in youth elite Cross-Country skiers and biathletes: A pilot study. Appl Sci. 2024;14. 10.3390/app14072774.

[5] Sandbakk O, Ettema G, Holmberg HC. Gender differences in endurance performance by elite cross-country skiers are influenced by the contribution from poling. Scand J Med Sci Sports. 2014;24(1):28–33. 22621157 10.1111/j.1600-0838.2012.01482.x

[6] Sunde A, et al. Stronger is better: the impact of upper body strength in double poling performance. Front Physiol. 2019;10:1091. 31507453 10.3389/fphys.2019.01091 PMC6716506

[7] American College of Sports. American college of sports medicine position stand. Progression models in resistance training for healthy adults. Med Sci Sports Exerc. 2009;41(3):687–708. 19204579 10.1249/MSS.0b013e3181915670

[8] Pearcey GEP, et al. Chronic resistance training: is it time to rethink the time course of neural contributions to strength gain? Eur J Appl Physiol. 2021;121(9):2413–22. 34052876 10.1007/s00421-021-04730-4

[9] Grgic J, Schoenfeld BJ. Are the hypertrophic adaptations to high and Low-Load resistance training muscle fiber type specific?? Front Physiol. 2018;9:402. 29720946 10.3389/fphys.2018.00402 PMC5915697

[10] Schoenfeld BJ, et al. Strength and hypertrophy adaptations between Low- vs. High-Load resistance training: A systematic review and Meta-analysis. J Strength Cond Res. 2017;31(12):3508–23. 28834797 10.1519/JSC.0000000000002200

[11] Warneke K, et al. Maximal strength measurement: A critical evaluation of common methods-a narrative review. Front Sports Act Living. 2023;5:1105201. 36873661 10.3389/fspor.2023.1105201 PMC9981657

[12] Benton MJ, Raab S, Waggener GT. Effect of training status on reliability of one repetition maximum testing in women. J Strength Conditioning Res, 2013. 27(7). 10.1519/JSC.0b013e3182752d4a 23037618

[13] Grgic J, et al. Test-Retest reliability of the One-Repetition maximum (1RM) strength assessment: a systematic review. Sports Med Open. 2020;6(1):31. 32681399 10.1186/s40798-020-00260-z PMC7367986

[14] McMaster DT, et al. A brief review of strength and ballistic assessment methodologies in sport. Sports Med. 2014;44(5):603–23. 24497158 10.1007/s40279-014-0145-2

[15] Benton MJ, Swan PD, Peterson MD. Evaluation of multiple one repetition maximum strength trials in untrained women. J Strength Cond Res. 2009;23(5):1503–7. 19620914 10.1519/JSC.0b013e3181b338b3

[16] Gonzalez-Badillo JJ, Sanchez-Medina L. Movement velocity as a measure of loading intensity in resistance training. Int J Sports Med. 2010;31(5):347–52. 20180176 10.1055/s-0030-1248333

[17] Eston R, Evans HJ. The validity of submaximal ratings of perceived exertion to predict one repetition maximum. J Sports Sci Med. 2009;8(4):567–73. 24149599 PMC3761544

[18] Flanagan E, Jovanović M. Researched applications of velocity based strength training. J Australian Strength Cond. 2014;22:58–69.

[19] Clemente FM et al. Validity and reliability of the inertial measurement unit for barbell velocity assessments: A systematic review. Sens (Basel), 2021. 21(7). 10.3390/s21072511 PMC8038306 33916801

[20] Wlodarczyk M et al. Effects of Velocity-Based training on strength and power in elite Athletes-A systematic review. Int J Environ Res Public Health, 2021. 18(10). 10.3390/ijerph18105257 PMC8156188 34069249

[21] Thompson SW, et al. Is it a slow day or a go day? The perceptions and applications of velocity-based training within elite strength and conditioning. Int J Sports Sci Coaching. 2022;18(4):1217–28.

[22] Pestana-Melero FL, et al. Reliability of the load-Velocity relationship obtained through linear and polynomial regression models to predict the 1-Repetition maximum load. J Appl Biomech. 2018;34(3):184–90. 29252060 10.1123/jab.2017-0266

[23] Banyard HG, et al. The reliability of individualized Load-Velocity profiles. Int J Sports Physiol Perform. 2018;13(6):763–9. 29140148 10.1123/ijspp.2017-0610

[24] Conceicao F, et al. Movement velocity as a measure of exercise intensity in three lower limb exercises. J Sports Sci. 2016;34(12):1099–106. 26395837 10.1080/02640414.2015.1090010

[25] Bazuelo-Ruiz B, et al. Predicting maximal dynamic strength from the Load-Velocity relationship in squat exercise. J Strength Cond Res. 2015;29(7):1999–2005. 25881572 10.1519/JSC.0000000000000821

[26] Appleby BB, et al. Validity and reliability of methods to determine barbell displacement in heavy back squats: implications for Velocity-Based training. J Strength Cond Res. 2020;34(11):3118–23. 33105362 10.1519/JSC.0000000000002803

[27] Orange ST, et al. Validity and reliability of a wearable inertial sensor to measure velocity and power in the back squat and bench press. J Strength Cond Res. 2019;33(9):2398–408. 29742745 10.1519/JSC.0000000000002574

[28] Orange ST, et al. Test-Retest reliability of a commercial linear position transducer (GymAware powerTool) to measure velocity and power in the back squat and bench press. J Strength Cond Res. 2020;34(3):728–37. 29952868 10.1519/JSC.0000000000002715

[29] Boehringer S, Whyte DG. Validity and Test-Retest reliability of the 1080 quantum system for bench press exercise. J Strength Cond Res. 2019;33(12):3242–51. 31136548 10.1519/JSC.0000000000003184

[30] García-Ramos A, et al. Assessment of the load-velocity profile in the free-weight prone bench pull exercise through different velocity variables and regression models. PLoS ONE. 2019;14(2):e0212085. 30811432 10.1371/journal.pone.0212085 PMC6392250

[31] Sanchez-Medina L, et al. Velocity- and power-load relationships of the bench pull vs. bench press exercises. Int J Sports Med. 2014;35(3):209–16. 23900903 10.1055/s-0033-1351252

[32] Loturco I, et al. Force-Velocity relationship in three different variations of prone row exercises. J Strength Cond Res. 2021;35(2):300–9. 29489715 10.1519/JSC.0000000000002543

[33] Torrejon A, et al. The load-velocity profile differs more between men and women than between individuals with different strength levels. Sports Biomech. 2019;18(3):245–55. 29558855 10.1080/14763141.2018.1433872

[34] Hautier CA, et al. Optimal velocity for maximal power production in non-isokinetic cycling is related to muscle fibre type composition. Eur J Appl Physiol Occup Physiol. 1996;74(1):114–8. 8891509 10.1007/BF00376503

[35] Larsson L, Moss RL. Maximum velocity of shortening in relation to myosin isoform composition in single fibres from human skeletal muscles. J Physiol. 1993;472:595–614. 8145163 10.1113/jphysiol.1993.sp019964 PMC1160504

[36] Sale DG. Influence of exercise and training on motor unit activation. Exerc Sport Sci Rev. 1987;15:95–151. 3297731

[37] Hakkinen K. Neuromuscular and hormonal adaptations during strength and power training. A review. J Sports Med Phys Fit. 1989;29(1):9–26. 2671501

[38] Behm DG. Neuromuscular implications and applications of resistance training. J Strength Conditioning Res, 1995. 9(4).

[39] Zhang M, et al. Comparison of velocity and Percentage-based training on maximal strength: Meta-analysis. Int J Sports Med. 2022;43(12):981–95. 35255509 10.1055/a-1790-8546

[40] Goldsmith JA, et al. Validity of the open barbell and tendo weightlifting analyzer systems versus the Optotrak certus 3D Motion-Capture system for barbell velocity. Int J Sports Physiol Perform. 2019;14(4):540–3. 30300064 10.1123/ijspp.2018-0684

[41] Hughes LJ, et al. Using a Load-Velocity relationship to predict one repetition maximum in Free-Weight exercise: A comparison of the different methods. J Strength Cond Res. 2019;33(9):2409–19. 31460988 10.1519/JSC.0000000000002550

[42] McKay AKA, et al. Defining training and performance caliber: A participant classification framework. Int J Sports Physiol Perform. 2022;17(2):317–31. 34965513 10.1123/ijspp.2021-0451

[43] Hopkins WG. Measures of reliability in sports medicine and science. Sports Med. 2000;30(1):1–15. 10907753 10.2165/00007256-200030010-00001

[44] Lorenz DS, et al. What performance characteristics determine elite versus nonelite athletes in the same sport? Sports Health. 2013;5(6):542–7. 24427430 10.1177/1941738113479763 PMC3806174

[45] Haff GGT. N.T., *Essentials of Strength Training and Conditioning, 4th Edition.* Medicine & Science in Sports & Exercise, 2016. 48(10).

[46] Schoenfeld BJ, et al. Differential effects of heavy versus moderate loads on measures of strength and hypertrophy in Resistance-Trained men. J Sports Sci Med. 2016;15(4):715–22. 27928218 PMC5131226

[47] Akca F. Prediction of rowing ergometer performance from functional anaerobic power, strength and anthropometric components. J Hum Kinet. 2014;41:133–42. 25114740 10.2478/hukin-2014-0041 PMC4120446

[48] Zatsiorsky VM, Kraemer WJ. Timing in strength training. In: science and practice of strength training. Champaign, IL: Human Kinetics; 2006.

[49] Feuerbacher JF, et al. Validity and Test-Retest reliability of the Vmaxpro sensor for evaluation of movement velocity in the deep squat. J Strength Cond Res. 2023;37(1):35–40. 36515587 10.1519/JSC.0000000000004207

[50] Riemann BL, Lininger MR. Statistical primer for athletic trainers: the essentials of Understanding measures of reliability and minimal important change. J Athl Train. 2018;53(1):98–103. 29332472 10.4085/1062-6050-503-16 PMC5800735

[51] Vetter TR, Schober P. Agreement analysis: what he said, she said versus you said. Anesth Analg. 2018;126(6):2123–8. 29677066 10.1213/ANE.0000000000002924

[52] Dorrell HF, et al. Validity and reliability of a linear positional transducer across commonly practised resistance training exercises. J Sports Sci. 2019;37(1):67–73. 29851551 10.1080/02640414.2018.1482588

[53] Banyard HG, et al. Validity of various methods for determining velocity, force, and power in the back squat. Int J Sports Physiol Perform. 2017;12(9):1170–6. 28182500 10.1123/ijspp.2016-0627

[54] Garnacho-Castano MV, Lopez-Lastra S, Mate-Munoz JL. Reliability and validity assessment of a linear position transducer. J Sports Sci Med. 2015;14(1):128–36. 25729300 PMC4306764

[55] McGrath G, et al. Velocity based training: validity of monitoring devices to assess mean concentric velocity in the bench press exercise journal of Australian strength and conditioning ASCA. Journal of Australian Strength and Conditioning ASCA; 2018. p. 26.

[56] Koo TK, Li MY. A guideline of selecting and reporting intraclass correlation coefficients for reliability research. J Chiropr Med. 2016;15(2):155–63. 27330520 10.1016/j.jcm.2016.02.012 PMC4913118

[57] Greig L, et al. The predictive validity of individualised Load-Velocity relationships for predicting 1RM: A systematic review and individual participant data Meta-analysis. Sports Med. 2023;53(9):1693–708. 37493929 10.1007/s40279-023-01854-9 PMC10432349

[58] Banyard HG, Nosaka K, Haff GG. Reliability and validity of the Load-Velocity relationship to predict the 1RM back squat. J Strength Conditioning Res. 2017;31(7):1897–904. 10.1519/JSC.0000000000001657 27669192

[59] Macarilla CT, et al. Accuracy of predicting One-Repetition maximum from submaximal velocity in the barbell back squat and bench press. J Hum Kinet. 2022;82:201–12. 36196346 10.2478/hukin-2022-0046 PMC9465738

[60] Loturco I, et al. Predicting the maximum dynamic strength in bench press: the high precision of the bar velocity approach. J Strength Cond Res. 2017;31(4):1127–31. 28328719 10.1519/JSC.0000000000001670

[61] Ruf L, Chery C, Taylor KL. Validity and reliability of the Load-Velocity relationship to predict the One-Repetition maximum in deadlift. J Strength Cond Res. 2018;32(3):681–9. 29466270 10.1519/JSC.0000000000002369

[62] Cotterman ML, Darby LA, Skelly WA. Comparison of muscle force production using the Smith machine and free weights for bench press and squat exercises. J Strength Cond Res. 2005;19(1):169–76. 15705030 10.1519/14433.1

[63] Cormie P, Deane R, McBride JM. Methodological concerns for determining power output in the jump squat. J Strength Cond Res. 2007;21(2):424–30. 17530961 10.1519/R-19605.1

[64] Crewther BT, et al. Validating two systems for estimating force and power. Int J Sports Med. 2011;32(4):254–8. 21380970 10.1055/s-0030-1270487

[65] Janicijevic D, et al. Bench press 1-Repetition maximum Estimation through the individualized Load-Velocity relationship: comparison of different regression models and minimal velocity thresholds. Int J Sports Physiol Perform. 2021;16(8):1074–81. 33771947 10.1123/ijspp.2020-0312

[66] Balsalobre-Fernandez C, Kipp K. Use of Machine-Learning and Load-Velocity profiling to estimate 1-Repetition maximums for two variations of the Bench-Press exercise. Volume 9. Sports (Basel); 2021. 3. 10.3390/sports9030039 PMC8002214 33809614

[67] Garcia-Ramos A. Optimal minimum velocity threshold to estimate the 1-Repetition maximum: the case of the Smith machine bench press exercise. Int J Sports Physiol Perform. 2023;18(4):393–401. 36791730 10.1123/ijspp.2022-0355

[68] Fitas A, et al. Optimal Minimum-Velocity threshold to predict One-repetition maximum for the back squat. Int J Sports Med. 2024;45(12):923–9. 39068935 10.1055/a-2335-4143

[69] Guppy SN, Kendall KL, Haff GG, Strength. & Conditioning Journal, 2024. 46(3).

